# Causal association between hyperthyroidism and risk of gastroesophageal reflux or esophageal cancer: a bidirectional Mendelian randomization investigation

**DOI:** 10.3389/fendo.2024.1411629

**Published:** 2024-09-17

**Authors:** Xingyu Zhu, Ming Li, Hanghang Gan, Yingqiang Guo

**Affiliations:** ^1^ Department of Cardiovascular Surgery, West China School of Medicine, West China Hospital, Sichuan University, Chengdu, China; ^2^ Cardiovascular Surgery Research Laboratory, West China Hospital, Sichuan University, Chengdu, China

**Keywords:** hyperthyroidism, esophageal cancer, gastroesophageal reflux, Mendelian randomization, European ancestry

## Abstract

**Background:**

Emerging observational studies indicated an association between hyperthyroidism and gastrointestinal disorders. However, it remains unclear whether this association is causal, particularly in the case of gastroesophageal reflux (GERD) and esophageal cancer.

**Methods:**

To assess the potential causal relationship between hyperthyroidism and GERD or esophageal cancer, we conducted a bidirectional 2-sample Mendelian randomization study. Independent genetic instruments for hyperthyroidism from the UK Biobank (N case=3,545 and N control=459,388) and public genome-wide association study (GWAS) dataset (N case=3,731 and N control=480,867) were used to investigate the association with esophageal cancer in the UK Biobank study (N case=740 and N control=372,016) and GERD in the public GWAS database (N case=20,381 and N control=464,217). Four different approaches (inverse variance weighted (IVW), weighted mode, MR-Egger, and weighted median regression) were used to ensure that our results more reliable. Additional sensitivity analyses were also performed to validate our results.

**Results:**

When hyperthyroidism was considered as the exposure factor, it appeared to act as a protective factor for GERD (OR_IVW_ = 0.88, 95% CI, 0.79-0.99, P = 0.039), while as a risk factor for esophageal cancer (OR_IVW_ = 1.03, 95% CI, 1.01-1.06, P = 0.003). However, there is no evidence supporting a reverse causal relationship between genetic susceptibility to hyperthyroidism and GERD or esophageal cancer.

**Conclusion:**

Our findings provided genetic evidence supporting bidirectional causal relationships between hyperthyroidism and GERD or esophageal cancer. These results substantiate certain discoveries from previous observational studies on a causal level and provide insight into relevant genetic susceptibility factors.

## Introduction

Thyroid hormones and their derivatives play a crucial role in regulating glucose, lipid, and cholesterol metabolism of key organs (1). Disruptions in thyroid hormone levels and alterations in related signaling pathways can lead to severe pathological conditions. Hyperthyroidism is characterized by the suppression of thyrotropin and elevated concentrations of triiodothyronine (T3) and/or free thyroxine (FT4), which affects approximately 2.5% of adults worldwide (2, 3). Untreated hyperthyroidism can lead to arrhythmias, mood disturbances, osteoporosis, depressive disorder, and metabolic disturbances such as increased resting energy expenditure and glucose generation (4, 5). In addition, hyperthyroidism may also induce gastrointestinal system dysfunction through various mechanisms. Hyperthyroid state can induce increased secretion of gastrin, pancreatic juice, and bile, as well as accelerated gastrointestinal motility, resulting in diarrhea, and some patients may experience steatorrhoea (6). Prior research has shown the potential impact of hyperthyroidism on cellular proliferation and differentiation, consequently elevating the risk of certain cancers (7). Additionally, high level of thyroid hormones may trigger the generation of anti-gastric parietal cell antibodies (PCA), leading to decreased gastric acid secretion and autoimmune gastritis (8).

Gastroesophageal reflux disease (GERD) affects approximately 20% of the adult population worldwide and is associated with an increased risk of esophagitis and esophageal cancer (9). Patients with GERD may experience heartburn, acid regurgitation, and other persistent symptoms, significantly impacting their quality of life and work (10). GERD is primarily caused by the reflux of strong gastric acid, and the standard medical approach for GERD involves medications aimed at reducing or neutralizing gastric acid secretion, such as proton pump inhibitors (PPIs) and aluminum hydroxide (11). Thus, the generation of PCA due to hyperthyroid state, leading to decreased gastric acid secretion, may serve as a potential protective factor against GERD. However, there is currently no reported research establishing a causal relationship between hyperthyroidism and GERD. Furthermore, esophageal cancer is the sixth most common cause of cancer-related death worldwide, posing a significant challenge to global health (12). Despite recent progress in esophageal cancer treatment, the five-year survival rate for patients remains relatively poor (13). Hence, further investigation is needed to explore the etiology of esophageal cancer and its potential risk factors.

The Mendelian randomization (MR) approach explores causal associations between exposure and outcome variables, with resistance to reverse causation or confounding factors (14). MR studies leverage the random allocation of genetic variations, the lack of reciprocal influence among distinct traits, and the constancy of allele frequencies in the presence of disease, thereby mitigating limitations associated with observational studies and randomized controlled trials (15, 16).

In this investigation, we utilized publicly accessible genome-wide association study (GWAS) data and performed a two-sample MR analysis to investigate the potential causal effects of high thyroid hormones level on GERD and esophageal cancer. Additionally, we conducted a reverse MR analysis to assess casual effects of GERD and esophageal cancer on hyperthyroidism to fully elucidate their interactions and provide new insights into underlying mechanisms.

## Method

### Study design and datasets

**Figure 1:**
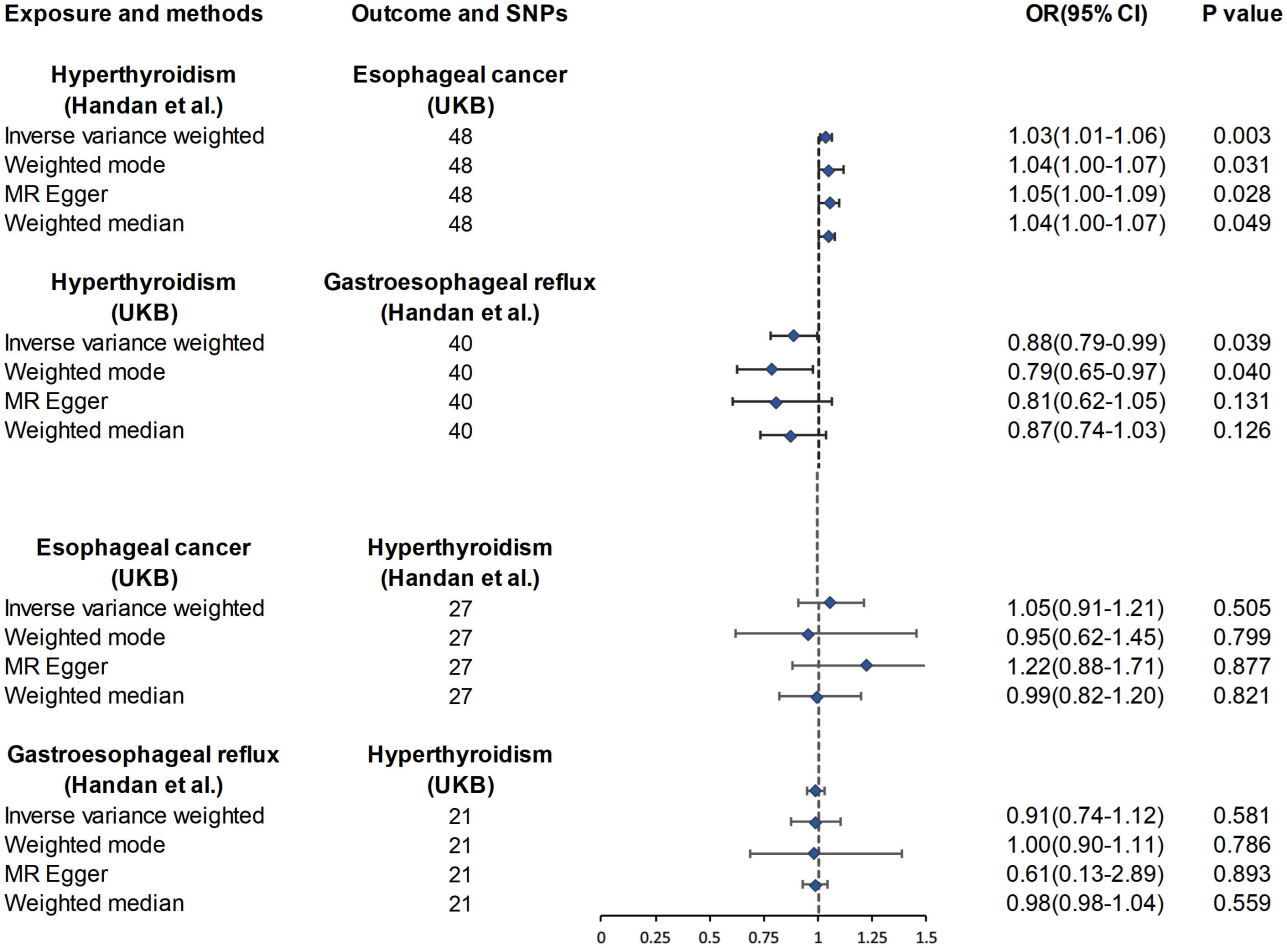
Association of genetic susceptibility to hyperthyroidism with Mendelian randomizations of esophageal cancer and GERD. CI, confidence interval; OR, odds ratio; UKB, UK Biobank; Statistical significance: p < 0.05.

We conducted a two-sample bidirectional MR study using summary data from different GWAS cohorts of European ancestry. The schematic representation of the bidirectional MR study design is illustrated in **Figure 1**. The primary data source for this investigation is presented in **Table 1**. Single-nucleotide polymorphisms (SNPs) employed in MR analyses to establish a causal effect must satisfy three core assumptions: (1) the genetic instruments should demonstrate a robust association with the exposure; (2) the SNPs should not be linked to any confounding factor influencing the risk factor-outcome relationship; (3) the SNPs should not influence the outcome through any pathway other than the target exposure. Establishing causality becomes challenging in the absence of fulfilling any of the aforementioned assumptions (17, 18).

**Table 1:** Details of studies included in the Mendelian randomization analyses for the association between hyperthyroidism and esophageal cancer or gastroesophageal reflux.

Trait	Database	Year	Consortium	Population	SNP	Sample size
Hyperthyroidism	Handan; Nat Aging	2021	NA	European	9,587,836	484,598
Hyperthyroidism	ukb-b-20289	2021	UKB	European	9,851,867	462,933
Esophageal cancer	ieu-b-4960	2021	UKB	European	8,970,465	372,756
Gastroesophageal reflux	Handan; Nat Aging	2021	NA	European	9,587,836	484,598

SNPs, single-nucleotide polymorphisms; UKB, UK Biobank; NA, Not applicable.

Genetic association data related to hyperthyroidism were obtained from the UK Biobank (UKB) and GWAS dataset published by Handan et al., with a sample size of 462,933 (N case=3,545 and N control=459,388) and 484,598 (N case=3,731 and N control=480,867), respectively (19). In order to perform two sample MR analysis between hyperthyroidism and esophageal cancer or GERD, we retrieved esophageal cancer data from UKB with a sample size of 372,756 (N case=740 and N control=372,016) and GERD data from the dataset of Handan et al. (19) with a sample size of 484,598 (N case=20,381 and N control=464,217) for a non-overlapping dataset with hyperthyroidism. Ethical approval is waived for the original GWAS study had previously obtained ethical clearance from their respective institutional review boards. Our investigation follows the guidelines outlined in the Strengthening the Reporting of Observational Studies in Epidemiology (STROBE) (20).

### Selection of instrumental variables

The selection of optimal instrumental variables (IVs) in this study adhered to high-quality criteria aimed at upholding the integrity and precision of the research. To meet the relevance assumption, SNPs associated with the respective exposure were selected below the genome-wide significance threshold of 1×10^-5^. The “clump” method was applied to select independent SNPs, characterized by a linkage disequilibrium (LD) r^2^ < 0.001 and a distance > 10,000 kb (21). Ensuring the accuracy of the MR analysis necessitated the exclusion of palindromic SNPs, defined as those with effect alleles and complementary alleles. Additionally, the strength of the association between each IV and the exposure were assessed. We computed the F-statistic (Beta^2^/SE^2^) for each SNP, ensuring that the F-statistic exceeded 10 to establish robust instrument validity (22). Ultimately, these rigorously selected SNPs were utilized as the final instrumental variables for subsequent MR analysis.

### Statistical analysis

To satisfy the foundational assumptions of MR analysis and provide a comprehensive evaluation of the causal association between exposures and outcomes, we utilized four distinct MR approaches: Inverse Variance Weighting (IVW), Weighted Median, Weighted Mode, and MR Egger, to estimate causal effects. IVW served as the primary analytical method, delivering the highest statistical power when all IVs were effective instruments (23). To assess the robustness of our MR analysis results, sensitivity analyses, including heterogeneity tests, pleiotropy tests, and leave-one-out analyses, were conducted and visualized. Heterogeneity of the selected SNPs was evaluated using the Cochran Q test. If the P-value from the heterogeneity test was <0.05, we utilized the MRPRESSO method to identify and remove IVs with heterogeneity from the analysis. No SNPs associated with confounding factors were identified in our study using PhenoScanner database (http://www.phenoscanner.medschl.cam.ac.uk/) (24). Additionally, the impact of pleiotropic SNPs on the MR analyses was assessed by examining the MR-Egger intercept (25). Furthermore, we performed a leave-one-out analysis to scrutinize the impact of individual SNPs on the overall causal relationship. Funnel plots were utilized to evaluate the symmetry of the selected SNPs, forest plots were employed to assess the reliability and heterogeneity of the associated estimates, and scatter plots were used to visually represent the relationship between exposure and outcome effects.

In the reverse analysis, we applied the same methods, utilizing a set of SNPs associated with esophageal cancer and GERD to examine the causal relationship with hyperthyroidism (**Figure 1**). Statistical analyses were conducted using R software (version 4.3.1, MR package).

## Results

### IVs selection

In the forward analysis, we obtained 48 and 40 IVs independent of linkage disequilibrium (LD) from hyperthyroidism. In the reverse analysis, 27 and 21 SNPs associated with esophageal cancer and GERD, respectively, were designated as instrumental variables. The F-statistical value for each selected instrumental variable surpasses 10, indicating a low likelihood of weak instrumental variable bias. Specifics regarding SNPs associated with exposure can be found in **Supplementary Table 1**.

### Causal association between hyperthyroidism and GERD

**Figure 2:**
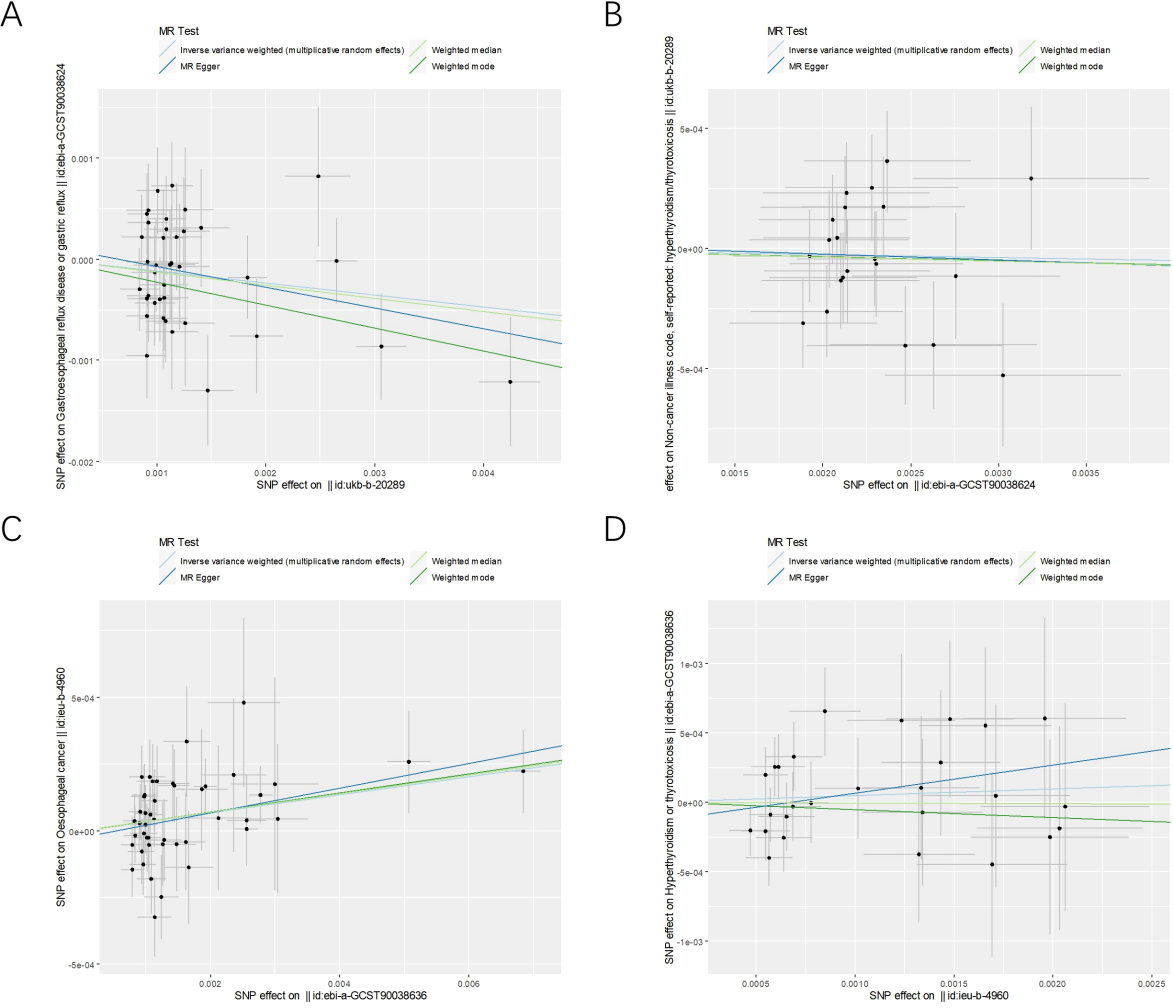
The scatter plots for the causal effect of forward and reverse MR analysis. **(A)** hyperthyroidism on GERD. **(B)** GERD on hyperthyroidism. **(C)** hyperthyroidism on esophageal cancer. **(D)** esophageal cancer on hyperthyroidism.

As shown in **Supplementary Table 1**, after excluding 3 and 2 heterozygous SNPs for hyperthyroidism and GERD in forward and reverse analysis respectively, we obtained a final set of 40 and 21 SNPs for each exposure in the analysis. Of note, hyperthyroidism served as a protective factor against the development of GERD (OR_IVW_ = 0.88, 95% CI, 0.79-0.99, P = 0.039; OR_Weighted mode_ = 0.79, 95% CI, 0.65-0.97, P=0.040; OR_MR-Egger_ = 0.81, 95% CI, 0.62-1.05, P = 0.131; OR_Weighted median_ = 0.87, 95% CI, 0.74-1.03, P = 0.126) (**Figure 1**). However, there was also no evidence for an association between GERD and hyperthyroidism in the reverse analyses. **Figures 2A, B** presented the relevant scatter plots, illustrating the effect sizes of SNPs for each phenotype in the bidirectional analysis.

### Causal association between hyperthyroidism and esophageal cancer

**Figure 1** exhibits the outcomes of the estimated causal associations between hyperthyroidism and esophageal cancer. In the forward and reverse analyses, 48 and 27 valid SNPs were extracted for hyperthyroidism and esophageal cancer, respectively. Notably, hyperthyroidism is correlated with an increasing risk of esophageal cancer occurrence (odds ratio (OR)_IVW_ = 1.03, 95% confidence interval (CI), 1.01-1.06, P = 0.003; OR_Weighted mode_ = 1.04, 95% CI, 1.00-1.07, P=0.031; OR_MR-Egger_ = 1.05, 95% CI, 1.00-1.09, P = 0.028; OR_Weighted median_ = 1.04, 95% CI, 1.00-1.07, P = 0.049). However, no significant associations could be found between esophageal cancer and hyperthyroidism in the reverse analyses (**Figure 1**). The relevant scatter plots depicting the effect sizes of SNPs for each phenotype in the bidirectional analysis were presented in **Figures 2C, D**.

### Sensitivity analysis

**Figure 3:**
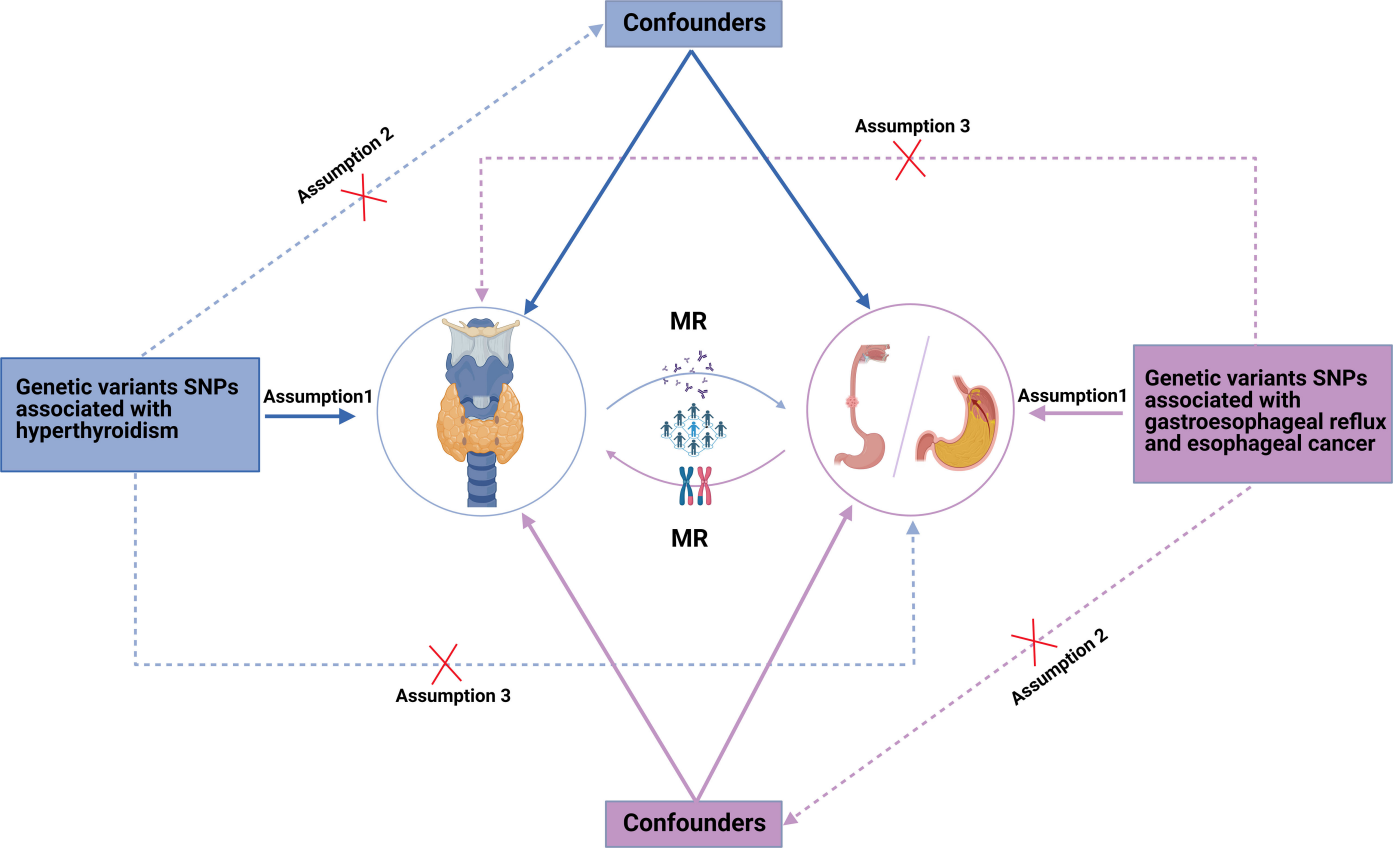
The overview of study design and assumptions of the Mendelian randomization design. Assumption 1: Instrumental variables are strongly associated with atopic dermatitis. Assumption 2: Instrumental variables are independent of any confounders. Assumption 3: Instrumental variables affect esophageal cancer susceptibility varying from different ancestry. MR, Mendelian randomization; SNPs, single-nucleotide polymorphisms.

The MR pleiotropy analysis and heterogeneity test indicated that, in the casual interaction between hyperthyroidism and GERD or esophageal cancer, neither the Cochran’s Q statistic nor the MR Egger regression intercept were significant (p > 0.05), signifying the absence of heterogeneity or horizontal pleiotropy (**Table 2**). Furthermore, the leave-one-out analysis and visualization results demonstrate the robustness of our findings. The relevant leave-one-out sensitivity plots, and funnel plots of the present study are shown in **Supplementary Figures 1**, **2**. We utilized the MRPRESSO method to identify and remove outlier IVs with heterogeneity from the analysis. The detailed information of the excluded SNPs has been listed in **Supplementary Tables 1**, **2**. The overview of study design and assumptions of the MR design was shown in **Figure 3**.

**Table 2:** Heterogeneity test and horizontal pleiotropy test.

Exposure	Outcome	Heterogeneity test			Horizontal pleiotropy test		
		Method	Cochran’s Q	Q_ pval	Egger_intercept	Se	pval
Hyperthyroidism (Handan et al.)	Esophageal cancer (UKB)	MR Egger	35.99	0.799	<0.001	<0.001	0.457
		IVW	36.55	0.811			
Esophageal cancer (UKB)	Hyperthyroidism (Handan et al.)	MR Egger	21.75	0.649	-0.001	0.001	0.324
		IVW	22.76	0.646			
Hyperthyroidism (UKB)	GERD (Handan et al.)	MR Egger	39.95	0.341	0.001	0.001	0.472
		IVW	40.52	0.359			
GERD (Handan et al.)	Hyperthyroidism (UKB)	MR Egger	39.95	0.341	0.001	0.001	0.619
		IVW	40.52	0.359			

IVW, inverse-variance weighted; GERD, gastroesophageal reflux; UKB, UK Biobank; MR, Mendelian randomization. Statistical significance: p < 0.05.

## Discussion

Currently, there are few studies exploring the potential causal and pathogenic associations between hyperthyroidism and esophageal cancer or GERD. This bidirectional two-sample MR study provides new insights for the causal associations between hyperthyroidism and esophageal cancer or GERD. Generally, GERD serves as a precursor to esophageal cancer. However, we found that genetic susceptibility to hyperthyroidism is associated with an increased risk of esophageal cancer, while acting as a protective factor against GERD. This seemingly contradictory result may unveil the diverse impact of excessive amounts of thyroid hormones on the digestive tract. Additionally, our reverse MR analysis indicates that genetic susceptibility to GERD or esophageal cancer appears unrelated to the risk of hyperthyroidism.

The etiology of hyperthyroidism is complex, presenting as a clinical syndrome caused by the overproduction of thyroid hormones (26). Hyperthyroidism patients experience elevated thyroid hormone levels and accelerated body metabolism, leading to abnormal gastric acid secretion, accelerated gastrointestinal motility, and a corresponding increase in bowel movements (27). Consequently, atypical gastrointestinal symptoms in hyperthyroid patients can lead to misdiagnosis and mistreatment. Excessive thyroid hormones levels may exert direct toxic effects on the liver, compounded by the increased metabolic demands leading to hepatic hypoxia and insufficient nutrient supply (28). This may result in abnormalities in blood biochemical indices, such as transaminase, alanine aminotransferase, and bilirubin, potentially leading to a misdiagnosis of viral hepatitis or other liver diseases. Therefore, further research employing innovative methodologies to investigate the causal association between hyperthyroidism and digestive disorders is warranted.

Circadian rhythms are intrinsic time mechanisms within organisms that regulate various physiological processes to maintain internal balance. Thyroid hormones play a crucial role in metabolism, thermoregulation, and growth, and their secretion is also influenced by circadian rhythms (29). Studies have shown that sleep deprivation inhibits thyroid hormone secretion, with nighttime sleep deprivation causing a sustained increase in thyroid hormone levels, resulting in significantly elevated morning TSH levels (30). Furthermore, circadian rhythms play an important role in regulating the occurrence and progression of GERD (31). Research indicates that GERD symptoms are more pronounced at night, which is related to fluctuations in nocturnal gastric acid secretion. Gastric acid secretion peaks during the night and early morning, leading to the lowest gastric pH and increasing the risk of acid reflux (29, 32). Additionally, night shift workers are at a significantly higher risk of GERD and/or erosive esophagitis compared to day shift workers, possibly due to circadian rhythm disruptions affecting gastric acid secretion and lower esophageal sphincter function (30). This suggests that hyperthyroidism may mitigate the impact of circadian rhythm disturbances on the development and progression of GERD. Elevated thyroid hormone levels in hyperthyroid patients may enhance gastrointestinal motility and reduce gastric acid secretion, thereby decreasing the likelihood of gastric content reflux (32). Moreover, hyperthyroidism may influence GERD incidence by affecting the pressure of the lower esophageal sphincter (LES). Research indicates that melatonin can increase LES pressure and reduce gastric acid secretion (31). In hyperthyroid patients, altered melatonin levels may indirectly modulate LES function, thereby reducing gastric content reflux.

In previous investigations, the association between hyperthyroidism and cancer risk has been widely explored. In a review study conducted by Tran et al. (33), the analysis revealed an increased risk of breast, prostate, and thyroid cancers in individuals with hyperthyroidism. A nested case-control study by Bursi et al. (34) indicated an increased risk of colorectal cancer associated with hyperthyroidism (adjusted OR = 1.21, 95% CI = 1.08 to 1.36, P = .001). Interestingly, similar risks were identified in untreated hypothyroidism (adjusted OR = 1.16, 95% CI = 1.08 to 1.24, P <.001). Ghalaut et al. (35) observed significantly elevated levels of FT3, FT4, T3, and T4 in patients with acute leukemia compared to non-leukemic controls, alongside lower TSH levels. These findings suggest that elevated thyroid hormone levels may stimulate cancer cell proliferation in specific tissues, potentially contributing to cancer initiation and progression. Similarly, in Mendelian randomization studies, Xu et al. (7) reported a positive correlation between hyperthyroidism and the risk of colorectal cancer, while the risk of prostate cancer showed a negative correlation. Additionally, previous research has indicated that thyroid hormone receptors (TRs), encoded by the TRα and TRβ genes, may have tumor-suppressive properties, suggesting that thyroid hormones could also act as protective factors against cancer (36). In the future, relevant molecular biology research and prospective randomized controlled studies could provide further evidence to verify these results.

Researchers have long explored the association between thyroid disorders and the pathophysiology of the digestive system, primarily focusing on gastric histology and gastric acid output capacity (37). Seino et al. (38) previously reported hypercoagulability in hyperthyroidism, speculating that the high reactivity of beta-adrenergic cells producing gastrin might be a mechanism for hyperthyroidism. Of note, previous findings indicated that in patients with hyperthyroidism, the positivity rate of anti-gastric parietal cell antibodies (PCA) is generally around 30%, significantly higher than age- and gender-matched controls (39). Irvine et al. (40) reported a common occurrence of gastric acid deficiency or low acidity in autoimmune thyroid disease, often associated with the high incidence and titers of PCA. The gastric mucosal damage caused by PCA and possible cell-mediated immunity may be significant reasons for reduced gastric acid secretion in patients with hyperthyroidism. Therefore, in hyperthyroid patients with gastric acid deficiency, the elevated serum gastrin levels are considered a feedback response. Hyperthyroidism and autoimmune gastritis fall under the spectrum of organ-specific autoimmune diseases, with antibodies against thyroid and gastric mucosa often coexisting in this spectrum. Furthermore, in obese stain chicken serum with an organ-specific autoimmune predisposition, concurrent detection of anti-gastric PCA and thyroid antibodies has been documented (41). PCA-positive patients may experience conditions such as pernicious anemia, autoimmune atrophic gastritis, and gastric cancer (42). Thus, this study confirmed a reduced risk of GERD in hyperthyroid patients, potentially linked to mechanisms involving decreased gastric acid secretion, and identified genetic susceptibility factors for further exploration in future research.

The strength of our study lies in the utilization of bidirectional two-sample MR analyses to investigate the potential causal relationship between hyperthyroidism and esophageal cancer or GERD. Additionally, the use of exposure and outcome data from different sources minimizes the impact of sample overlap. However, inherent limitations exist in this study. First, the exploration of specific thyroid hormones in our bidirectional causal relationship with esophageal cancer or GERD was limited due to constraints in available SNPs data within the database. Secondly, despite employing an MR design and controlling for known confounding factors, undisclosed potential confounders may still affect the results. Thirdly, this study focused just on individuals of European ancestry, and since autoimmune diseases are generally more prevalent in females than males, stratification by gender was not possible due to data limitations (43). Finally, the etiology and various subtypes of hyperthyroidism are complex. However, due to the lack of SNP data for the relevant subtypes, analysis is currently not feasible, which is a limitation of our MR analysis.

## Conclusion

Our study employed bidirectional two-sample MR analyses to investigate the potential causal association between hyperthyroidism and esophageal cancer or GERD. When hyperthyroidism was considered as the exposure factor, we found that hyperthyroidism might act as a protective factor for GERD. This association may be linked to the production of anti-gastric PCA in hyperthyroid patients, leading to reduced gastric acid secretion. However, hyperthyroidism was identified as a risk factor for esophageal cancer. Further research is warranted to explore this association’s underlying pathological and physiological mechanisms.

## Data Availability

The original contributions presented in the study are included in the article/**Supplementary Material**. Further inquiries can be directed to the corresponding author.
