# Supplementary material for: Causal association between hyperthyroidism and risk of gastroesophageal reflux or esophageal cancer: a bidirectional Mendelian randomization investigation
Source: Front Endocrinol (Lausanne). 2024 Sep 17;15:1411629. doi: 10.3389/fendo.2024.1411629 (PMC11442246; doi:10.3389/fendo.2024.1411629)
Supplement: Supplementary file 1 [file DataSheet1.docx]

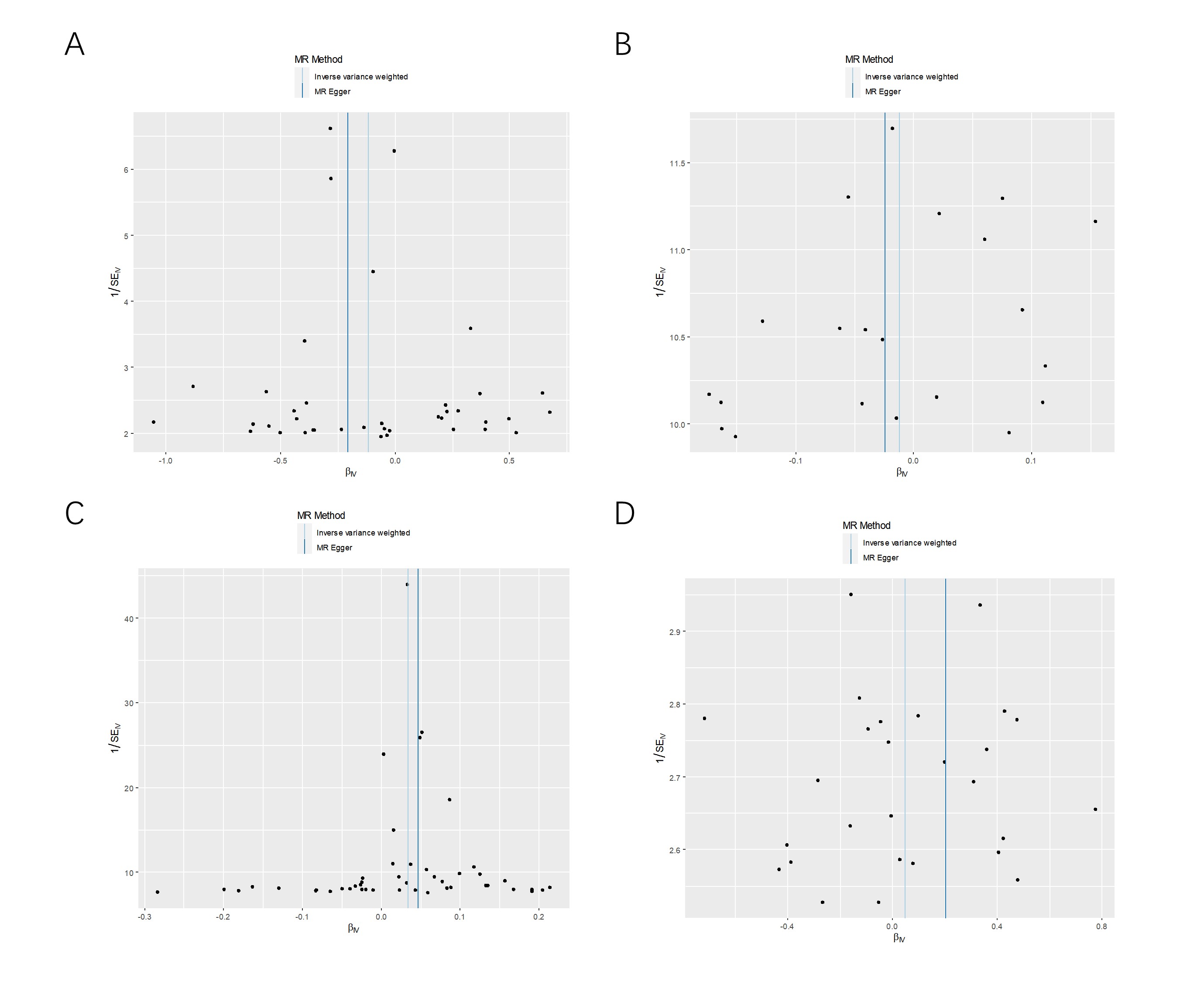


**Supplementary Fig.1** The funnel plots for the causal effect of forward and reverse MR analysis. (A) hyperthyroidism on GERD. (B) GERD on hyperthyroidism. (C) hyperthyroidism on esophageal cancer. (D) esophageal cancer on hyperthyroidism.
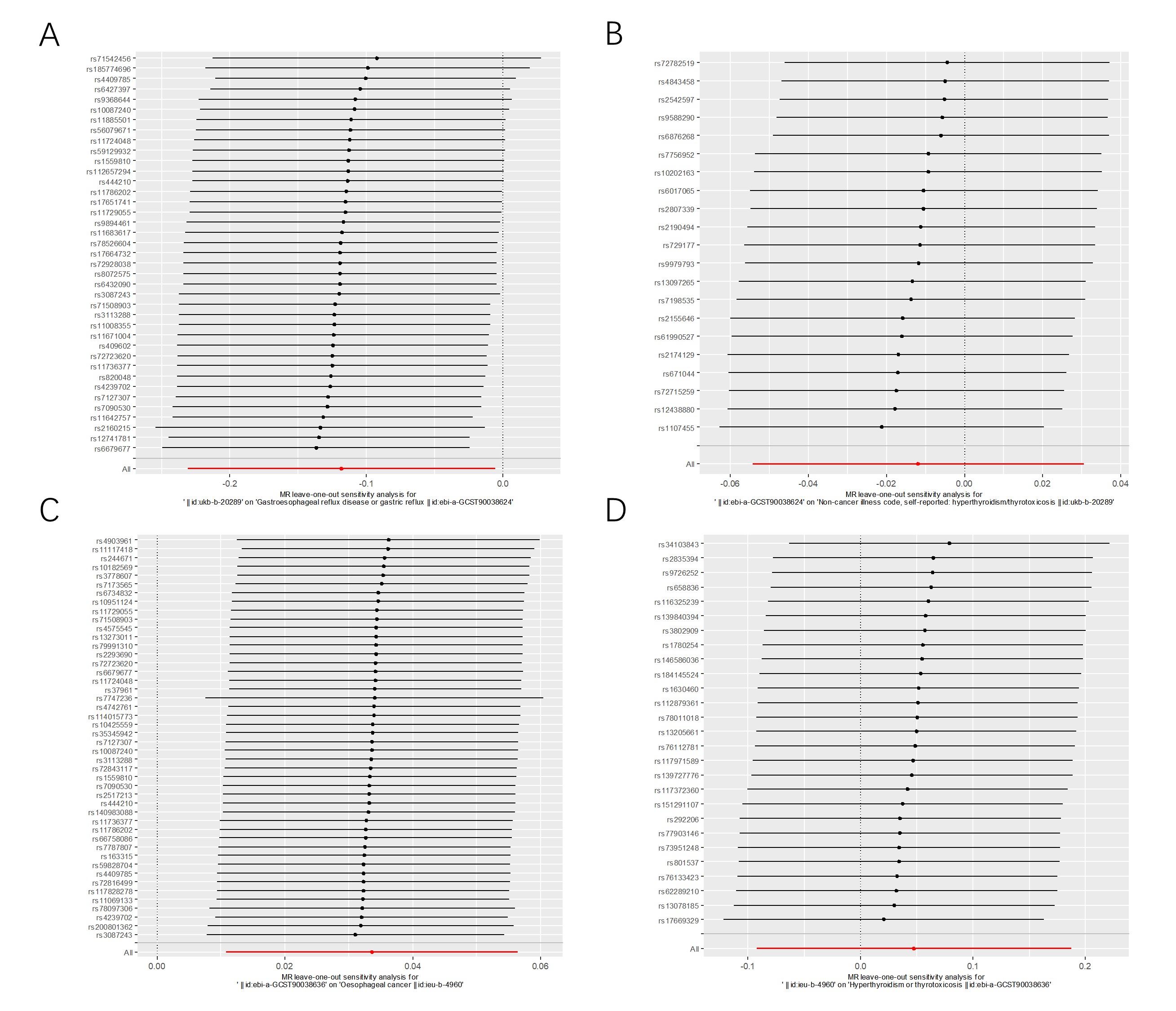


**Supplementary Fig.2** The leave-one-out plots for the causal effect of forward and reverse MR analysis. (A) hyperthyroidism on GERD. (B) GERD on hyperthyroidism. (C) hyperthyroidism on esophageal cancer. (D) esophageal cancer on hyperthyroidism.
